# Extraosseous Ewing Sarcoma in Children: A Systematic Review and Meta-Analysis of Clinicodemographic Characteristics

**DOI:** 10.3390/children9121859

**Published:** 2022-11-29

**Authors:** Maher Ghandour, Burkhard Lehner, Matthias Klotz, Andreas Geisbüsch, Jakob Bollmann, Tobias Renkawitz, Axel Horsch

**Affiliations:** 1 Department of Orthopedics, Heidelberg University Hospital, 69129 Heidelberg, Germany; 2 Orthopedics and Trauma Surgery, Marienkrankenhaus Soest, 59494 Soest, Germany

**Keywords:** Ewing sarcoma, extraosseous, children

## Abstract

**Background:** We conducted this systematic review to provide comprehensive evidence on the prevalence, clinical features and outcomes of young extraosseous Ewing sarcoma (EES) cases. **Methods:** PubMed, Scopus, Web of Science, and Google Scholar were searched for articles reporting the occurrence of EES among children and adolescents (<21 years). The primary outcome included the rate of occurrence of EES among children and adolescents, while the secondary outcomes included the descriptive analyses of the demographic characteristics, tumor characteristics, and clinical outcomes of the affected cases. The data are reported as the effect size (ES) and its corresponding 95% confidence interval (CI). **Results:** A total of 29 studies were included. Twenty-four reported instances of childhood disease among all the EES cases [ES = 30%; 95%CI: 29–31%], while five studies reported extraosseous cases among the pediatric EES cases [ES = 22%; 95%CI: 13–31%]. The thorax is the most common location of childhood EES [33%; 95%CI: 20–46%] followed by the extremities [31%; 95%CI: 22–40%]. Concurrent chemotherapy and radiotherapy [57%; 95%CI: 25–84%] was the most commonly implemented management protocol in the pediatric EES cases. The rate of no evidence of disease and 5-year overall survival was 69% for both outcomes. Mortality occurred in 29% of cases, while recurrence and secondary metastasis occurred in 35% and 16% of cases, respectively. **Conclusions:** Our findings provide insight into the clinical features and outcomes of EES among children and adolescents.

## 1. Introduction

The Ewing sarcoma family of tumors (ESFT) is a collection of small, rounded tumor cells that have similar neural histological and genetic characteristics [1,2,3,4]. ESFT is categorized into four types based on the origin of the tumor: Ewing sarcoma of the bone, peripheral primitive neuroectodermal tumor (pPNET), Askin tumor, which originates from the chest wall, and, finally, the extraosseous or extraskeletal Ewing sarcoma (EES). EES, which occurs in around 20% of ES cases, typically originates from the soft tissues of the trunk and extremities [5], and the majority of these cases are reported among patients who are 10–30 years of age [6].

Based on a previous report, the incidence of EES is 0.4 per million individuals, which is lower than that of ES of the bone by 10-fold [7]. Although uncommon, the occurrence of EES seems to have a bimodal distribution, where there is a peak in the occurrence rate among children (<5 years) and adults (>35 years) [8], with an increased likelihood of presenting among older populations compared to ES of the bone. Unlike Ewing earcoma of the bone, no evidence supports a link between the tumor and race or biological sex [8,9,10].

The management of EES includes surgery [11] and chemotherapy [10,12,13] in resectable tumors. Under unresectable conditions, radiotherapy is usually considered [14]. According to the National Comprehensive Cancer Network (NCCN), the optimum management of EES remains not clearly defined [15,16], although some studies have highlighted an added value of surgery among EES cases compared to Ewing sarcoma of the bone in terms of better survival rates [17,18]. In general, the prognosis of EES is more favorable than that of the bone [9,10].

To date, there is no clear picture regarding the occurrence rate of EES among children and adolescents (<21 years), as well as their demographic characteristics, tumor characteristics (i.e., location), treatment modalities, and clinical outcomes (i.e., survival, mortality, recurrence). Therefore, we conducted this systematic review and meta-analysis to provide collective evidence regarding the clinical characteristics and outcomes in this patient population.

## 2. Materials and Methods

### 2.1. Study Design and Search Strategy

This systematic review was performed in accordance with the Preferred Reporting Items for Systematic Reviews and Meta-Analyses (PRISMA) guidelines [19]. A protocol was not registered, since it is not mandatory, as per several recommendations [20,21]. On 26 July 2022, PubMed, Scopus, Web of Science, and Google Scholar were searched for articles that report the presentation of EES in the pediatric population (children and young adolescents <21 years of age). Of note, only the first 200 records from Google Scholar were retrieved and screened according to recently published guidelines [22]. We updated the database search on 24 August 2022 to ensure that no additional relevant reports had been published prior to the qualitative and quantitative analyses [23].

We used a combination of keywords and terms in our search, which included the following: (“Ewing Sarcoma” OR “Ewing’s Sarcoma”) AND (adolescen* OR Child* OR Pediatric* OR “young adult”) AND (“soft tissue” OR extraskeletal OR extraosseous) AND (clinicopathologic* OR “clinical feautre” OR “clinical characteristic*” OR “clinical outcome*”). The terms of the Medical Subject Headings (MeSH) were also added (particularly in PubMed) to retrieve all the possibly relevant articles. The detailed search criteria used for each database are described in Supplementary Table S1.

Moreover, we conducted a manual search to find any relevant articles that may have potentially been excluded during the screening phase or were not found during the database search [24,25]. This strategy was conducted through three different approaches: (1) screening the titles of the reference list of the final included papers, (2) reading the titles and abstracts of articles similar to final included studies through the “similar articles” function on PubMed, and (3) conducting a random search on Google using keywords similar to those of the original database search, such as: “Ewing sarcoma” + “child”. It is noteworthy that no filters were used during the database search regarding the language of the research paper, year in which the paper was published, or the country of the first author.

### 2.2. Eligibility Criteria

The methodology and design of this review were conducted as per the PICO framework [26,27], including the population (pediatric cases of EES), intervention (none), comparison (none), and outcome (primary outcome: prevalence rate of EES in children and adolescents; secondary outcomes: clinicodemographic characteristics, tumor characteristics, and clinical outcomes in pediatric cases of EES). 

For articles to be included, a study had to: (1) report original data, (2) include cases of EES, (3) report cases aged <21 years. On the other hand, studies were excluded if they were compliant with one of the following criteria: (1) non-original research (i.e., review articles, editorials without human data, commentaries, theses, conference abstracts/posters, and books), (2) animal, in vivo, and in vitro studies, (3) case reports and case series of <5 cases, (4) studies reporting EES cases of mixed ages (children, adolescents, adults, and elderly) without stratifying the cases according to their age, (5) studies reporting Ewing sarcomas of mixed origin (extraosseous and skeletal) in children without stratifying the cases according to their origin, and (6) duplicated records. 

### 2.3. Screening and Study Selection

Following the retrieval of records through the database search [28], the references were imported to EndNote (Version 8) for duplicate removal and to organize the screening sheet [29]. The screening sheet included the following: article ID, list of authors’ names, year of publication (YOP), research paper’s title, DOI, journal name, and abstract. The screening was carried out in three separate stages: title, abstract, and full-text screening. All of these steps were performed by two sets of two reviewers each. Any differences between the reviewers were reviewed and resolved by the senior author [30].

Significantly, upon reviewing the literature, two categories of articles were found to be consistent with our eligibility criteria. The first group of articles included patients with EES, among whom pediatric cases were counted, and the second group of articles included pediatric cases, of whom the origin of Ewing sarcoma was determined (extraosseous or skeletal). Both of these categories were included, extracted, and presented separately in our review.

### 2.4. Extraction and Quality Assessment

The data extraction process was carried out in a similar manner as the screening stage [31]. The senior author designed a pilot data extraction sheet through the Excel software (version 2021) that was consistent with the study objectives. The sheet included 5 domains. The first domain highlighted the baseline characteristics of the included studies (authors’ names, year of publication, country, study design, sample size, and follow-up duration). The second domain included the demographic characteristics of the included participants, such as age and biological sex. The third domain included the location of the EES among the pediatric cases (i.e., cranium, female genital tract, orbit, head and neck, pelvis, extremities, thorax, abdomen). The fourth domain included the tumor’s characteristics (i.e., management modalities (i.e., surgery alone, surgery combined with radiotherapy, surgery combined with chemotherapy, etc.). The final domain included the patients’ clinical outcomes in terms of the overall survival (OS), progression-free survival (PFS), disease-specific survival (DSS), secondary metastasis, no evidence of disease (NED), mortality, and recurrence. Two reviewers extracted the data from the included studies for further qualitative and quantitative synthesis, as per the recommended guidelines [32,33].

### 2.5. Data Synthesis

All quantitative analyses were conducted using the STATA software (version 17) with the metaprop command [34]. The exact cimethod [34] was used to pool the effect size (ES)—occurrence rate of EES in the pediatric cases—along with its 95% confidence interval (CI). Importantly, for the purposes of discussing the findings of our review, the term ES will refer to the effect size and not Ewing sarcoma (which will not be abbreviated in this manuscript). The random-effects and fixed-effects models were used according to the presence or absence of heterogeneity, respectively [35,36]. Heterogeneity was measured using the *I*^2^ statistic, where a value of >50% or a *p*-value of <0.05 indicates significant heterogeneity. 

## 3. Results

### 3.1. Search Results

**Figure 1:**
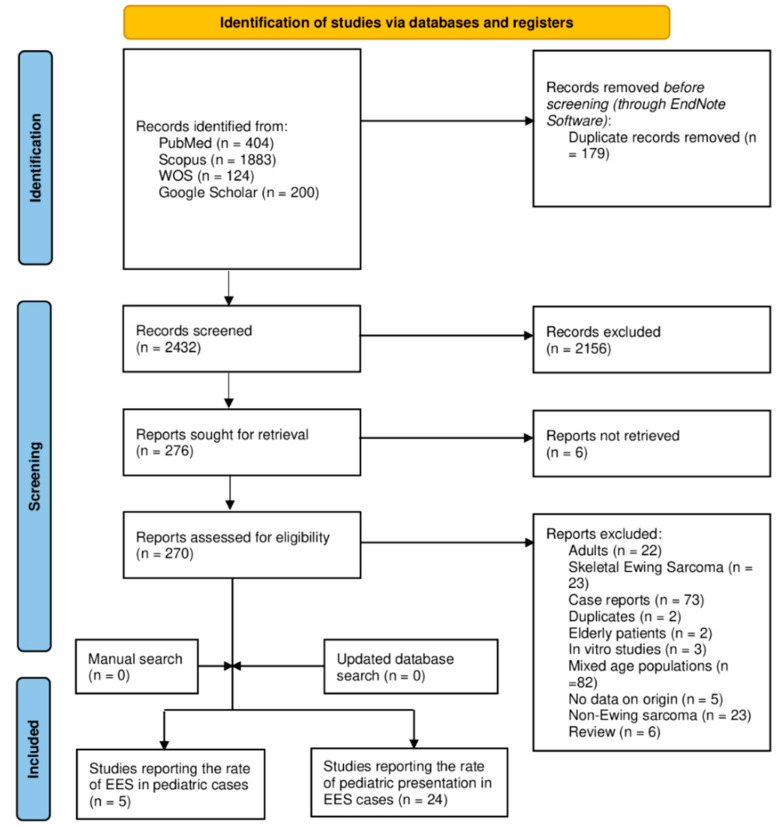
A PRISMA diagram showing the database search and screening results of the review.

A summary of the results of the electronic database search, as well as the screening stage, is provided in Figure 1. The initial database search resulted in 2611 references, out of which 179 duplicated records were found and removed using the EndNote software (version 8). The titles and abstracts of 2432 articles were screened, resulting in 276 articles eligible for full-text screening. The full texts of six studies were not found and, therefore, these were excluded. A total of 241 studies were excluded as follows: adult cases (n = 22), skeletal involvement (n = 23), case reports (n = 73), duplicated records (n = 2), elderly cases (n = 2), in vitro studies (n = 3), mixed-age populations (n = 82), no data on sarcoma origin (n = 5), non-Ewing sarcoma (n = 23), and reviews (n = 6). The updated and manual search yielded no more studies, so that the final number of included studies was equal to 29 reports. Twenty-four articles reported the rate of childhood cases among those with EES (of all ages), while five studies reported the rate of extraosseous involvement in pediatric Ewing sarcoma (of mixed origin—skeletal and extraosseous) cases. 

### 3.2. Baseline Characteristics of the Included Studies

The baseline characteristics of the included studies are presented in Table 1. Among the 24 studies that reported the rate of childhood cases among all the EES cases (adults and children), two were conducted in the United Kingdom (UK), two in China, one in Germany, three in India, three in Italy, one in Japan, one in Korea, one in Turkey, and ten in the United States (US). Five studies were case series, fourteen were retrospective chart reviews, one was a registry-based study, three were SEER-based studies, and one was a secondary analysis of three prospective studies. The number of included patients with EES in the individual studies ranged from 8 [37,38,39] to as high as 3178 [40] patients, with a total sample size of 5752 patients with EES. The follow-up was reported in 16 studies, ranging from as low as 0.9 [41] months to as high as 349 months [42].

Among the five studies that reported the rate of EES among children with ES, one study was conducted in the Netherlands, three in the US, and one in China. Three studies were retrospective chart reviews, one was a multicenter cohort study, and one was a secondary analysis of two clinical trials. The sample size of included pediatric patients with Ewing sarcoma ranged from 18 [43] to 1039 cases [9], with an overall sample size of 1190. The follow-up duration was reported in only two studies ranging from 56.4 [44] to 120 months [45].

**Table 1:** Baseline characteristics of the included studies.

Author (YOP)	Country	Design	Sample	FU (Months)
**Studies reporting the rate of childhood among all EES Cases (adults and children)**				
Banerjee (1997) [37]	UK	Case series	8	5–12
Biswas (2014) [46]	India	Chart review	374	25 *
Boyce-Fappiano (2021) [2]	USA	Chart review	60	74 *
Casanova (2007) [47]	Italy	Case series	52	120 *
Chen (2019) [48]	USA	Chart review	31	24.8
Chiang (2017) [49]	USA	Chart review	19	NR
Deshpande (2021) [38]	India	Case series	8	15–43
Gupta (2010) [50]	USA	Chart review	53	46.8 *
Jiang (2018) [40]	China	SEER-based study	3178	NR
Koka (2021) [39]	USA	Case series	8	52.63
Koscielniak (2021) [4]	Germany	Secondary analysis of three prospective studies	243	84 *
Lee (2010) [51]	Korea	Chart review	94	24.9
Livellara (2022) [42]	Italy	Chart review	57	5–349
Muratori (2020) [52]	Italy	Chart review	29	37 *
Murugan (2018) [53]	USA	Chart review	23	5–156
Pradhan (2011) [54]	UK	Chart review	253	87
Qureshi (2013) [55]	India	Chart review	32	NR
Raney (1997) [56]	USA	Registry-based study	130	NR
Neriman (2009) [57]	Turkey	Case series	13	NR
Takenaka (2016) [58]	Japan	Chart review	74	44 *
Tarek (2020) [41]	USA	Chart review	30	0.9
Verma (2017) [59]	USA	SEER-based study	415	NR
Wong (2015) [60]	USA	SEER-based study	550	NR
Xie (2010) [61]	China	Chart review	18	NR
**Studies reporting the rate of EES among cases of childhood Ewing sarcoma**				
Bosma (2022) [45]	The Netherlands	Multicenter cohort	60	120
Cash (2015) [9]	USA	Secondary analysis of 2 clinical trials	1039	NR
Huh (2017) [44]	USA	Chart review	42	56.4 *
Majeed (2019) [62]	USA	Chart review	31	NR
Xiao (2016) [43]	China	Chart review	18	NR

* Data are reported as the median and not the mean. YOP: year of publication; USA: United States of America; NR: not reported; UK: United Kingdom; FU: follow-up; EES: extraosseous Ewing sarcoma.

### 3.3. Demographic Characteristics of the Included Participants

The demographic characteristics of the included patients are illustrated in Table 2. Among the studies that included patients with EES regardless of their age, the rate of affected children ranged from as low as 5.63% (31 out of 550 cases) [60] to as high as 100% [55,56]. The pooled rate of childhood EES among the patients with EES was 30% [3001 patients, 95%CI: 29–31%; I^2^ = 99.01%]. Out of the pediatric cases diagnosed with EES, 52.45% were males (224 out of 427 patients).

Among the studies that included pediatric cases of Ewing sarcoma regardless of its origin, the rate of presentation with a disease of extraosseous origin ranged from 10% (6 out of 60 cases) [45] to 55.55% (10 out of 18 cases) [42], with an overall pooled rate of 22% [1190 patients, 95%CI: 13–31%; I^2^ = 19.28%]. Out of the pediatric cases diagnosed with EES, 54.70% (122 out of 223) were males.

### 3.4. The Location of EES in the Pediatric Cases

Among the included studies, only 13 reported data regarding the location of EES among the pediatric cases (Table 3). The pooled meta-analysis revealed that the thorax was the predominant site where EES occurred [33%; 95%CI: 20–46%] followed by the extremities [31%; 95%CI: 22–40%], the head and neck [14%; 95%CI: 7–21%], the pelvis [13%; 95%CI: 9–16%], the abdomen [10%; 95%CI: 4–16%], the spine [8%; 95%CI: 6–11%], the intracranial space [8%; 95%CI: 1–33%], and finally the orbit [2%; 95%CI: 0–4%]. Of note, among the pediatric cases, the occurrence of EES in the skin, the kidney, and the female genital tract was scarcely reported, and the performance of a meta-analysis was not feasible due to the lack of sufficient data.

### 3.5. The Characteristics of EES among the Pediatric Cases

Among the included studies, the management modalities in childhood EES were described and reported in ten studies (Table 4), among which concurrent chemotherapy and radiotherapy [13 patients, 57%; 95%CI: 25–84%] was the most frequently employed treatment protocol, followed by surgery combined with radiotherapy [236 patients, 55%; 95%CI: 28–82%], surgery alone [223 patients, 53%; 95%CI: 37–68%], surgery combined with chemotherapy [36 patients, 29%; 95%CI: 5–52%], and finally radiotherapy alone [219 patients, 16%; 95%CI: 11–21%].

### 3.6. The Clinical Outcomes of EES among the Pediatric Cases

The clinical outcomes associated with childhood EES are presented in Table 5. The 5-year OS was reported in 11 studies, out of which 664/1066 pediatric EES cases survived. The pooled 5-year OS rate was 69% [95%CI: 56–81%]. The 5-year PFS, DSS, and DMFS were reported in only a single study, which was not enough to derive conclusions or be for the data to be included in a meta-analysis. Seven studies reported no evidence of disease among 257 out of the 288 cases, with a pooled rate of 69% [95%CI: 51–87%]. Morality was reported in ten studies, where 120 deaths occurred among 404 pediatric cases of EES, with a pooled mortality rate of 29% [95%CI: 25–33%]. Meanwhile, recurrence was reported in five studies (19 cases out of 60 pediatric EES cases), with a pooled recurrence rate of 35% [95%CI: 16–54%]. Finally, secondary metastasis was reported in three studies, occurring in 38 cases out of 236 pediatric EES patients, with a pooled rate of 16% [95%CI: 11–21%].

## 4. Discussion

There is limited evidence regarding the occurrence rate and clinical characteristics of EES in children. Our systematic review is the first to comprehensively discuss the prevalence, clinical features, and outcomes of EES patients of pediatric age (less than 21 years). A summary of our key findings can be found in Table 6. Overall, a total of 29 studies reporting on 5752 patients were analyzed. In our study, we found that the rate of affected children and adolescents with EES in a population with EES (mixed age) varied substantially between the studies, ranging from 5.63% [60] to as high as 100% [55]. This discrepancy could be related to the design and methodology of the included studies, since some studies included patients with EES regardless of the age group at baseline, while a few studies included pediatric cases of EES at baseline [55,56]. Overall, our meta-analysis revealed that 30% of the EES cases occurred among children and adolescents. Consistent with previous observations [8,9], no link was noted between EES presentation in children and biological sex. The pooled rate of male pediatric patients with EES was 52.45%, which is relatively similar to that of female cases (47.55%).

In addition, five studies included children affected with ES at baseline, and then the origin of the tumor was analyzed in these cases. The rate of EES out of all the ES types ranged from 10% to 55.55% among the individual studies. Again, the difference in reported rates could be related to the design and methodology implemented in each study. That being said, in our meta-analysis, the rate of EES occurrence among the pediatric ES cases was 22%, of whom 54.70% were males.

Data on the location of EES among pediatric cases is scarce, since the majority of the available studies in the literature include patients with mixed ages and tend to stratify the outcomes (i.e., survival) based on age (children vs. adults or the elderly), without stratifying the clinical characteristics or tumor characteristics based on the age of the examined patients. Therefore, the data reported in our review regarding the EES location in the pediatric cases rely mainly on case series with a case-by-case description of the tumor characteristics. Thirteen studies reported relevant data on the location of EES, and our pooled meta-analysis revealed that the thorax is the most predominant origin for EES in children and adolescents, followed by the extremities, the head and neck, the pelvis, the abdomen, the spine, and the intracranial space, respectively. In certain cases, the EES originated in the orbit among the pediatric cases; however, the occurrence rate did not surpass the rare event assumption (>5%). Additionally, other sites, such as the great toe [63], the mesocolon [64], the frontal sinus [65], and the penis [66], have been described as rare cases. Moreover, the kidneys [41,53], the skin [37], and the female genital tract [49] have been reported as sites of origin of EES in pediatric cases in several case series; however, not enough data were present to perform a meta-analysis of the prevalence in this case.

There is a debate on the best management approach for EES cases occurring in children, and this uncertainty is related to the rarity of EES, the discrepancy in its clinical presentation, and the differences in the patients’ characteristics [67]. In addition, this patient population is underrepresented in clinical trials directed towards the investigation of the efficacy and safety of various treatment modalities among pediatric cases of EES. In our review, only ten studies reported the treatment modalities according to different age groups, and the majority of the data were pooled from case series. Overall, the majority of cases were treated with concurrent chemotherapy and radiotherapy (57% of cases), followed by surgery and radiotherapy (55%), surgery alone (53%), surgery and chemotherapy (29%), and radiotherapy alone in cases of unresectable tumors (16%). It is important to mention that the confidence interval of these reported rates is wide, reflecting the imprecision of the reported effect estimates. Therefore, these data should be interpreted with caution and should not be perceived as representative of the EES pediatric population. More data from properly designed research studies are still needed to confirm this observation. Additionally, the available data did not present survival outcomes stratified by these treatment modalities in the pediatric cases separately. Therefore, future studies should carefully consider stratifying data (clinical characteristics and outcomes) based on the origin of the tumor (skeletal vs. extraskeletal) and age of the included patients (children vs. adults vs. the elderly).

In our study, we found that a great proportion of pediatric EES patients have a preferable prognosis in terms of their 5-year overall survival (with an overall rate of 69%), which is consistent with that of cases with no evidence of disease following treatment (an overall rate of 69%). However, mortality was documented in almost one-third of the pediatric EES population (120 deaths out of 404 cases, an overall rate of 29%). Additionally, recurrence was reported in 35% of cases, while secondary metastasis was reported in 16%. That being said, these rates should be based on the available data of 11 studies out of the 29 studies included in our review. Therefore, the presented data are not generalizable to the whole EES pediatric population.

Meanwhile, our review has several limitations. The most important is the fact that our estimates regarding the prevalence of childhood EES among EES cases (of all ages) or the prevalence of cases of extraosseous origin among the pediatric Ewing sarcoma cases could be overestimated, since the majority of the included studies investigated EES cases and not the Ewing sarcoma population as a whole. In addition, most of these studies are based on retrospective analyses and not cross-sectional in design, which further limits the generalizability of our findings.

## 5. Conclusions

Although it is difficult to draw solid conclusions, our results highlight the proportion of children affected by extraosseous Ewing sarcoma, with a special focus on the demographic characteristics, tumor characteristics, and clinical outcomes of the affected patients.

## Figures and Tables

**Table 2:** The demographic characteristics of the included participants in each study with an overall estimation of the rate of the presentation with EES in childhood.

Author (YOP)	Rate of Pediatric Cases in EES				Biological Sex (Male)		
	N	T	%	Definition	N	T	%
**Studies reporting the rate of childhood among all EES cases (adults and children)**							
Banerjee (1997) [37]	6	8	75.00%	9–17	3	6	50%
Biswas (2014) [46]	29	60	48.33%	≤15	-	-	-
Boyce-Fappiano (2021) [2]	14	60	23.33%	≤20	-	-	-
Casanova (2007) [47]	9	9	100.00%	1–18	-	-	-
Chen (2019) [48]	17	31	54.83%	<20	-	-	-
Chiang (2017) [49]	4	19	21.05%	12–16	0	4	0%
Deshpande (2021) [38]	6	8	75.00%	1–13	2	6	33.33%
Gupta (2010) [50]	2	29	6.89%	0.3–16.2	-	-	-
Jiang (2018) [40]	413	981	42.09%	0–19	-	-	-
Koka (2021) [39]	4	8	50.00%	2–8	4	4	100%
Koscielniak (2021) [4]	221	243	90.94%	1–21	118	221	53.39%
Lee (2010) [51]	21	94	22.34%	≤12	-	-	-
Livellara (2022) [42]	18	57	31.57%	≤12	-	-	-
Muratori (2020) [52]	20	29	68.96%	<20	-	-	-
Murugan (2018) [53]	4	23	17.39%	8–19	2	4	50%
Pradhan (2011) [54]	70	129	54.26%	<16	-	-	-
Qureshi (2013) [55]	32	32	100.00%	1–19	23	32	71.87%
Raney (1997) [56]	130	130	100.00%	1–20	61	130	46.92%
Neriman (2009) [57]	13	13	100.00%	-	7	13	53.84%
Takenaka (2016) [58]	3	25	12.00%	-	-	-	-
Tarek (2020) [41]	7	30	23.33%	1–19	4	7	57.14%
Verma (2017) [59]	164	415	39.51%	0–18	-	-	-
Wong (2015) [60]	31	550	5.63%	<12 months	-	-	-
Xie (2010) [61]	9	18	50.00%	<18	-	-	-
**Total**	1247	3001	ES = 0.30 [95%CI: 0.29–0.31]		224	427	52.45%
**Author (YOP)**	**Rate of EES in Pediatric Cases**				**Biological sex (Male)**		
	**N**	**T**	**%**		**N**	**T**	**%**
**Studies reporting the rate of EES among cases of childhood Ewing sarcoma**							
Bosma (2022) [45]	6	60	10.00%	-	-	-	-
Cash (2015) [9]	213	1039	20.50%	-	116	213	54.46%
Huh (2017) [44]	6	42	14.28%	-	-	-	-
Majeed (2019) [62]	10	31	32.25%	-	-	-	-
Xiao (2016) [43]	10	18	55.55%	-	6	10	60.00%
**Total**	245	1190	ES = 0.22 [95%CI: 0.13–0.31]		122	223	54.70%

YOP: year of publication, N: number, T: total sample size, EES: extraosseous Ewing sarcoma.

**Table 3:** The location of extraosseous Ewing sarcoma in the pediatric cases.

Author (YOP)	Total	Skin—Subcutaneous Tissue (N)	Kidney (N)	Intra-cranial (N)	Female Genital Tract (N)	Orbit (N)	H&N (N)	Pelvis (N)	Spine (N)	Thorax (N)	Abdomen (N)	Extremity (N)
Banerjee (1997) [37]	6	6	-	-	-	-	-	-	-	-	-	-
Biswas (2014) [46]	-	-	-	-	-	-	-	-	-	-	-	-
Boyce-Fappiano (2021) [2]	-	-	-	-	-	-	-	-	-	-	-	-
Casanova (2007) [47]	-	-	-	-	-	-	-	-	-	-	-	-
Chen (2019) [48]	17	-	-	17	-	-	-	-	-	-	-	-
Chiang (2017) [49]	4	-	-		4	-	-	-	-	-	-	-
Deshpande (2021) [38]	6	-	-	6	-	-	-	-	-	-	-	-
Gupta (2010) [50]	-	-	-	-	-	-	-	-	-	-	-	-
Jiang (2018) [40]	-	-	-	-	-	-	-	-	-	-	-	-
Koka (2021) [39]	4	-	-	-	-	4	-	-	-	-	-	-
Koscielniak (2021) [4]	221	-	-	-	-	-	40	26	23	48	24	65
Lee (2010) [51]	-	-	-	-	-	-	-	-	-	-	-	-
Livellara (2022) [42]	-	-	-	-	-	-	-	-	-	-	-	-
Muratori (2020) [52]	-	-	-	-	-	-	-	-	-	-	-	-
Murugan (2018) [53]	4	-	4	-	-	-	-	-	-	-	-	-
Pradhan (2011) [54]	-	-	-	-	-	-	-	-	-	-	-	-
Qureshi (2013) [55]	32	-	-	-	-	-	11	-	-		1	19
Raney (1997) [56]	130	-	-	-	-	2	8	-	-	32	20	26
Neriman (2009) [57]	13	-	-	1	-	3		-	-	-	-	5
Takenaka (2016) [58]	-	-	-	-	-	-	-	-	-	-	-	-
Tarek (2020) [41]	7	-	7	-	-	-	-	-	-	-	-	-
Verma (2017) [59]	-	-	-	-	-	-	-	-	-	-	-	-
Wong (2015) [60]	-	-	-	-	-	-	-	-	-	-	-	-
Xie (2010) [61]	-	-	-	-	-	-	-	-	-	-	-	-
Bosma (2022) [45]	-	-	-	-	-	-	-	-	-	-	-	-
Cash (2015) [9]	213	-	-	-	-	-	17	29	15	92	-	56
Huh (2017) [44]	-	-	-	-	-	-	-	-	-	-	-	-
Majeed (2019) [62]	-	-	-	-	-	-	-	-	-	-	-	-
Xiao (2016) [43]	10	-	-	-	-	-	2	1	0	6	-	0
**Total**	N/T	6/6	11/11	24/36	4/4	9/147	78/606	56/444	38/444	178/574	45/383	171/619
	%	100	100	66.66	100	6.12	12.87	12.61	8.55	31.01	11.75	27.62
**ES [95%CI]**		N/A	N/A	0.08 [0.01–0.33]	N/A	0.02 [0.0–0.04]	0.14 [0.07–0.21]	0.13 [0.09–0.16]	0.08 [0.06–0.11]	0.33 [0.20–0.46]	0.10 [0.04–0.16]	0.31 [0.22–0.40]

YOP: year of publication; N: number of cases; T: total number of pediatric cases of EES; EES: extraosseous Ewing sarcoma; H&N: head and neck; ES: effect size; CI: confidence interval.

**Table 4:** Trends in the management modalities of pediatric cases of extraosseous Ewing sarcoma reported in the literature.

Author (YOP)	Surgery Alone		RT Alone		Surgery + RT		Surgery + CT		Concurrent CT with RT	
	N	T	N	T	N	T	N	T	N	T
Banerjee (1997) [37]	-	-	-	-	-	-	3	6	-	-
Biswas (2014) [46]	-	-	-	-	-	-	-	-	-	-
Boyce-Fappiano (2021) [2]	-	-	-	-	-	-	-	-	-	-
Casanova (2007) [47]	-	-	-	-	6	9	-	-	6	6
Chen (2019) [48]	-	-	-	-	-	-	-	-	-	-
Chiang (2017) [49]	3	4	-	-	1	4	0	4	-	-
Deshpande (2021) [38]	-	-	-	-	5	6	1	6	-	-
Gupta (2010) [50]	-	-	-	-	-	-	-	-	-	-
Jiang (2018) [40]	-	-	-	-	-	-	-	-	-	-
Koka (2021) [39]	-	-	-	-	3	4	-	-	-	-
Koscielniak (2021) [4]	-	-	-	-	-	-	-	-	-	-
Lee (2010) [51]	-	-	-	-	-	-	-	-	-	-
Livellara (2022) [42]	-	-	-	-	-	-	-	-	-	-
Muratori (2020) [52]	-	-	-	-	-	-	-	-	-	-
Murugan (2018) [53]	-	-	-	-	-	-	-	-	-	-
Pradhan (2011) [54]	-	-	-	-	-	-	-	-	-	-
Qureshi (2013) [55]	-	-	-	-	-	-	-	-	-	-
Raney (1997) [56]	-	-	-	-	-	-	-	-	-	-
Neriman (2009) [57]	-	-	-	-	-	-	13	13	-	-
Takenaka (2016) [58]	-	-	-	-	-	-	-	-	-	-
Tarek (2020) [41]	-	-	-	-	-	-	7	7	4	7
Verma (2017) [59]	-	-	-	-	-	-	-	-	-	-
Wong (2015) [60]	-	-	-	-	-	-	-	-	-	-
Xie (2010) [61]	-	-	-	-	-	-	-	-	-	-
Bosma (2022) [45]	4	6	1	6	-	-	-	-	-	-
Cash (2015) [9]	99	213	34	213	63	213	-	-	-	-
Huh (2017) [44]	-	-	-	-	-	-	-	-	-	-
Majeed (2019) [62]	-	-	-	-	-	-	-	-	-	-
Xiao (2016) [43]	-	-	-	-	-	-	-	-	-	-
Total	106	223	35	219	78	236	24	36	10	13
%	47.53%		15.98%		33.05%		66.67%		76.92%	
ES [95%CI]	0.53 [0.37–0.68]		0.16 [0.11–0.21]		0.55 [0.28–0.82]		0.29 [0.05–0.52]		0.57 [0.25–0.84]	

YOP: year of publication; CT: chemotherapy; RT: radiotherapy; ES: effect size; CI: confidence interval; N: number of cases; T: total sample of pediatric cases of EES; EES: extraosseous Ewing sarcoma.

**Table 5:** The clinical outcomes of pediatric patients with extraosseous Ewing sarcoma.

Author (YOP)	5-Year OS		5-Year PFS		5-Year DSS		5-Year DMFS		Mortality		NED		Recurrence		Secondary Metastasis	
	N	T	N	T	N	T	N	T	N	T	N	T	N	T	N	T
Banerjee (1997) [37]	-	-	-	-	-	-	-	-	1	6	-	-			1	6
Biswas (2014) [46]	16	29	11	29	-	-	-	-	-	-	-	-	-	-	-	-
Boyce-Fappiano (2021) [2]	-	-	-	-	12	14	9	14	-	-	9	14	-	-	-	-
Casanova (2007) [47]	9	9	-	-	-	-	-	-	1	9	-	-	-	-	1	9
Chen (2019) [48]	-	-	-	-	-	-	-	-	-	-	-	-	-	-	-	-
Chiang (2017) [49]	-	-	-	-	-	-	-	-	1	4	2	4	-	-	-	-
Deshpande (2021) [38]	-	-	-	-	-	-	-	-	1	6	4	6	2	6	-	-
Gupta (2010) [50]	-	-	-	-	-	-	-	-	-	-	-	-	-	-	-	-
Jiang (2018) [40]	268	413	-	-	-	-	-	-	-	-	-	-	-	-	-	-
Koka (2021) [39]	-	-	-	-	-	-	-	-	1	4	3	4	-	-	-	-
Koscielniak (2021) [4]	73	221	-	-	-	-	-	-	65	221	209	221	-	-	36	221
Lee (2010) [51]	16	21	-	-	-	-	-	-	-	-	-	-	-	-	-	-
Livellara (2022) [42]	17	18	-	-	-	-	-	-	-	-	-	-	-	-	-	-
Muratori (2020) [52]	14	20	-	-	-	-	-	-	-	-	-	-	-	-	-	-
Murugan (2018) [53]	-	-	-	-	-	-	-	-	1	4	-	-	1	4	-	-
Pradhan (2011) [54]	-	-	-	-	-	-	-	-	-	-	-	-			-	-
Qureshi (2013) [55]	26	32	-	-	-	-	-	-	-	-	29	32	6	32	-	-
Raney (1997) [56]	61	87	-	-	-	-	-	-	42	130	-	-	-	-	-	-
Neriman (2009) [57]	-	-	-	-	-	-	-	-	4	13	-	-	7	11	-	-
Takenaka (2016) [58]	2	3	-	-	-	-	-	-	-	-	-	-	-	-	-	-
Tarek (2020) [41]	-	-	-	-	-	-	-	-	3	7	1	7	3	7	-	-
Verma (2017) [59]	-	-	-	-	-	-	-	-	-	-	-	-	-	-	-	-
Wong (2015) [60]	-	-	-	-	-	-	-	-	-	-	-	-	-	-	-	-
Xie (2010) [61]	-	-	-	-	-	-	-	-	-	-	-	-	-	-	-	-
Bosma (2022) [45]	-	-	-	-	-	-	-	-	-	-	-	-	-	-	-	-
Cash (2015) [9]	162	213	-	-	-	-	-	-	-	-	-	-	-	-	-	-
Huh (2017) [44]	-	-	-	-	-	-	-	-	-	-	-	-	-	-	-	-
Majeed (2019) [62]	-	-	-	-	-	-	-	-	-	-	-	-	-	-	-	-
Xiao (2016) [43]	-	-	-	-	-	-	-	-	-	-	-	-	-	-	-	-
Total	664	1066	11	29	12	14	9	14	120	404	257	288	19	60	38	236
%	62.28%		37.93%		85.71%		64.28%		29.70%		89.23%		31.66%		16.10%	
ES [95%CI]	0.69 [0.56–0.81]		N/A		N/A		N/A		0.29 [0.25–0.33]		0.69 [0.51–0.87]		0.35 [0.16–0.54]		0.16 [0.11–0.21]	

YOP: year of publication; N: number of cases of the outcome; T: total number of pediatric cases of EES; EES: extraosseous Ewing sarcoma; ES: effect size; CI: confidence interval; OS: overall survival; PFS: progression-free survival; DSS: disease-specific survival; NED: no evidence of disease; DMFS: distant-metastatic free survival; N/A: not applicable for meta-analysis.

**Table 6:** Summary of the key findings on the pediatric EES cases in our review.

Outcome	Category	The Rate of Children among Patients with EES	The Rate of Extraosseous Origin in Pediatric Cases of ES
**Prevalence**			
	N/T	1247/3001	245/1190
	% [95%CI]	30% [29–31%]	22% [13–31%]
**Biological sex**			
	N/T	224/427	122/223
	%	52.45%	54.70%
**Location of EES in pediatric cases**			
**Most common**	Thorax	33% [20–46%]	
	Extremity	31% [22–40%]	
**Least common**	Orbit	2% [0–4%]	
**Clinical outcomes**			
	5-year OS	69% [56–81%]	
	Mortality	29% [25–33%]	
	NED	69% [51–87%]	
	Recurrence	35% [16–54%]	
	2ry metastasis	16% [11–21%]	

## Data Availability

The data presented in this manuscript can be provided by the corresponding author upon reasonable request.

## Supplementary Materials

The following supporting information can be downloaded at: https://www.mdpi.com/article/10.3390/children9121859/s1, Table S1: The detailed search strategy employed in each electronic database.
