# Supplementary material for: Extraosseous Ewing Sarcoma in Children: A Systematic Review and Meta-Analysis of Clinicodemographic Characteristics
Source: Children (Basel). 2022 Nov 29;9(12):1859. doi: 10.3390/children9121859 (PMC9776445; doi:10.3390/children9121859)
Supplement: Supplementary file 1 [file children-09-01859-s001.zip › children-2004235-Table S1.pdf]

Table S1. the detailed search strategy employed in each electronic database.

Database	No.	Search Query	Results
**PubMed [August 24, 2022]**			
	#1	"Ewing sarcoma" OR "Ewing's sarcoma" OR “Ewing family of tumors” OR “Ewing tumor” OR "Sarcoma, Ewing"[Mesh]	11048
	#2	Adolescen* OR Child* OR “Young adult*” OR Pediatric* OR Paediatric* OR "Pediatrics"[Mesh] OR "Adolescent"[Mesh] OR "Child"[Mesh] OR "Young Adult"[Mesh] OR "Adult Children"[Mesh]	5015527
	#3	“soft tissue” OR extraskeletal OR extra-skeletal OR extraosseous OR extra-osseous OR "Soft Tissue Neoplasms"[Mesh] OR "Neoplasms, Connective and Soft Tissue"[Mesh]	373630
	#4	Clinicopathologic* OR “clinical feature*” OR “clinical characteristic*” OR “clinical outcome*” OR “clinical presentation”	584569
	#5	#1 AND #2 AND #3 AND #4	404
**Scopus [August 24, 2022]**			
	#1	ALL ("Ewing sarcoma") OR ALL ("Ewing's sarcoma") OR ALL (“Ewing family of tumors”) OR ALL (“Ewing tumor”)	44723
	#2	ALL (Adolescen*) OR ALL (Child*) OR ALL (“Young adult*”) OR ALL (Pediatric*) OR ALL (Paediatric*)	10971188
	#3	ALL (“soft tissue”) OR ALL (extraskeletal) OR ALL (extra-skeletal) OR ALL (extraosseous) OR ALL (extra-osseous)	545307
	#4	ALL (Clinicopathologic*) OR ALL (“clinical feature*”) OR ALL (“clinical characteristic*”) OR ALL (“clinical outcome*”) OR ALL (“clinical presentation”)	3057044
	#5	#1 AND #2 AND #3 AND #4	1883
**Web of Science [August 24, 2022]**			
	#1	ALL="Ewing sarcoma" OR ALL="Ewing's sarcoma" OR ALL=“Ewing family of tumors” OR ALL=“Ewing tumor”	9388
	#2	ALL=Adolescen* OR ALL=Child* OR ALL=“Young adult*” OR ALL=Pediatric* OR ALL=Paediatric*	4134918
	#3	ALL=“soft tissue” OR ALL=extraskeletal OR ALL=extra-skeletal OR ALL=extraosseous OR ALL=extra-osseous	139564
	#4	ALL=clinicopathologic* OR ALL=“clinical feature*” OR ALL=“clinical characteristic*” OR ALL=“clinical outcome*” OR ALL=“clinical presentation”	613009
	#5	#1 AND #2 AND #3 AND #4	124
**Google Scholar [August 24, 2022]**			
	With all of the words	Ewing sarcoma child clinical	
	With the exact phrase		
	With at least one of the words	extraskeletal "soft tissue"	
	Total	Only the first 200 records were retrieved and screened	200
